# PREVALENCE OF INSULIN RESISTANCE AND ASSOCIATION WITH METABOLIC RISK FACTORS AND FOOD CONSUMPTION IN ADOLESCENTS - RECIFE/BRAZIL

**DOI:** 10.1590/1984-0462/2020/38/2019016

**Published:** 2020-03-16

**Authors:** Maria Izabel Siqueira de Andrade, Juliana Souza Oliveira, Vanessa Sá Leal, Niedja Maria da Silva Lima, Phelipe Bibiano Bezerra, Emerson Rogério Costa Santiago, Pedro Israel Cabral de Lira

**Affiliations:** a Universidade Federal de Pernambuco, Recife, PE, Brazil.; b Centro Universitário Maurício de Nassau, Recife, PE, Brazil.

**Keywords:** Insulin resistance, Overweight, Food consumption, Adolescent, Resistência à insulina, Sobrepeso, Consumo alimentar, Adolescente

## Abstract

**Objective:**

To identify the prevalence of insulin resistance in adolescents and its associations with metabolic factors and food intake.

**Methods:**

Cross-sectional study conducted with a stratified, complex, school-based sample. The subjects were adolescents (n=1,081) who participated in the Study of Cardiovascular Risk in Adolescents in the city of Recife (Pernambuco, Brazil). We analyzed demographic, socioeconomic, behavioral, anthropometric, biochemical, and dietary variables. Insulin resistance was defined as HOMA-IR>75^th^ percentile. A Poisson multivariate regression model with robust variance adjustment was used, and variables with p≤0.05 in the final model were considered statistically associated with insulin resistance.

**Results:**

Median age was 14 years (interquartile range: 13-16 years), and 25.3% of the sample showed insulin resistance. The variables associated with insulin resistance in the final model were age, body mass index-for-age (BMI/A), biochemical markers (triglycerides and high-density lipoprotein cholesterol) and saturated fat intake, with insulin resistance being more prevalent in individuals whose consumption of this type of fat was below the median of the sample distribution.

**Conclusions:**

Insulin resistance was prevalent in the adolescents analyzed and was significantly associated with metabolic variables and saturated fat intake.

## RESUMO

**Objetivo:**

Identificar a prevalência de resistência à insulina em adolescentes e verificar sua associação com variáveis metabólicas e com o consumo alimentar.

**Métodos:**

Estudo transversal, de base escolar, com amostra do tipo estratificada e complexa. Os indivíduos analisados foram adolescentes (n=1.081) participantes do Estudo de Riscos Cardiovasculares em Adolescentes (ERICA) na cidade de Recife (Pernambuco, Brasil). Foram coletadas variáveis demográficas, socioeconômicas, comportamentais, antropométricas, bioquímicas e do consumo alimentar. A resistência à insulina foi definida como HOMA-IR>percentil 75. Análises de regressão de Poisson com ajuste robusto da variância foram empregadas, sendo identificadas associações estatisticamente significativas quando p≤0,05.

**Resultados:**

A idade mediana foi de 14 anos (intervalo interquartílico=13-16) e a resistência à insulina foi evidenciada em 25,3% da amostra. As variáveis que se associaram significativamente com a resistência à insulina no modelo final foram a faixa etária, o índice de massa corpórea por idade (IMC/I), marcadores bioquímicos (triglicerídeos e HDL-colesterol) e o consumo alimentar de gordura saturada, observando-se maior prevalência da resistência à insulina naqueles indivíduos que apresentaram a ingestão deste tipo de gordura abaixo da mediana da própria distribuição.

**Conclusões:**

A resistência à insulina foi prevalente nos adolescentes avaliados e se associou significativamente com variáveis metabólicas e com o consumo alimentar de gordura do tipo saturada.

## INTRODUCTION

The epidemic trend and gradual increase in the prevalence of overweight and obesity in the Brazilian population are linked to a new profile of morbidity and mortality, in which there is a higher incidence of chronic non-communicable diseases even in the early stages of life.^1^ ^,^ ^2^ During adolescence, the bodily changes that are inherent to growth and sexual maturation may lead to excessive weight gain. This situation constitutes a primary risk factor for the development of insulin resistance (IR), which is a phenomenon that plays a major role in the emergence of cardiometabolic disorders.^3^

Dietary components can also influence insulin sensitivity. Evidence indicates a positive association between IR and the excessive intake of energy-dense foods rich in simple carbohydrates and fats, especially saturated and trans fats.^3^ ^,^ ^4^

Considering the methods proposed for detecting IR, the Homeostatic Model Assessment of Insulin Resistance (HOMA-IR) is the most used in epidemiological studies for being a fast, easy, low-cost method that effectively replaces the most sophisticated diagnostic techniques for IR.^5^

Thus, the present study aimed to identify the prevalence of IR in adolescents and its association with metabolic variables and food consumption.

## METHOD

This is a school-based, cross-sectional study conducted with adolescents aged 12 to 17 years enrolled in the last three years of elementary school or high school at public and private institutions in a Brazilian city located in Northeastern Brazil. The subjects were part of the Study of Cardiovascular Risks in Adolescents (ERICA - *Estudo de Riscos Cardiovasculares em Adolescentes*), which was a national multicenter study that aimed to estimate the prevalence of factors associated with cardiovascular risk in adolescents.^6^

This study complied with the guidelines outlined in the Declaration of Helsinki and was approved by the Human Research Ethics Committee of the Federal University of Pernambuco (certificate number: 05185212.2.2002.5208). The adolescents and their legal guardians were informed about all procedures, risks, and benefits of the study and agreed to participate by signing the informed consent form.

A three-stage stratified sampling method was used to calculate the sample size: school, class, and students, selected with probability proportional to size. In the chosen schools, a survey was performed of classes and students in the target age range. Three classes were selected per school, and all students from the chosen classes were asked to participate in the study. Other studies have published details on the ERICA sampling process.^6^ ^,^ ^7^

As the national survey generated representative data for the country, different regions, and major cities, we decided to evaluate a sub-sample of the total population analyzed in the ERICA study. Thus, the present investigation included only adolescents enrolled in the morning period of 39 public and private schools in the city of Recife, obtaining a sample of 1,081 adolescents.

Demographic, socioeconomic, and behavioral data on the adolescents were collected with the aid of a Personal Digital Assistant (PDA). Properly trained researchers gathered information pertinent to nutritional status, biochemical markers, and food consumption. We investigated the following demographic and socioeconomic variables: gender, age, ethnicity, and maternal schooling. The individuals were also categorized according to socioeconomic status using the criteria of the Brazilian Market Research Association,^8^ and the results were dichotomized as high socioeconomic status (classes A1, A2, B1, and B2) or low socioeconomic status (classes C, D, and E).

For the evaluation of nutritional status, weight was measured on an electronic scale with capacity for 200 kg and precision of 50 g. Height was measured in duplicate using a portable stadiometer with precision of 0.1 cm, assuming a maximum variation of 0.5 cm between the two measurements and calculating the mean. The reference standard to classify weight and height measures was that recommended by the World Health Organization (WHO),^9^ adopting the following cut-off points for categorizing the results: body mass index-for-age (BMI/A) Z score<1 - non-overweight individuals; BMI/A Z scores≥1 and <2 - overweight individuals, and Z score≥2 - obese individuals. Waist circumference (WC) was calculated using a non-elastic measuring tape placed horizontally at the midpoint between the lower edge of the last rib and the iliac crest. WC values ≥90^th^ percentile of distribution were considered for the diagnosis of abdominal obesity.^10^ The waist-to-height ratio (WHtR) was calculated using the WC and height, with values equal to or greater than 0.5 established as the cut-off point for abdominal obesity.^11^

Plasma glucose was measured using the GOD-PAP method in the modular analyzer Roche equipment. The lipid profile included the determination of total cholesterol, high-density lipoprotein cholesterol (HDL-C), and triglycerides (TG), which were analyzed using the colorimetric enzymatic method in the modular analyzer Roche equipment. Low-density lipoprotein cholesterol (LDL-C) was calculated by the Friedewald formula.^12^ Plasma insulin was determined with immunoassays. Serum lipids were classified following the recommendations of the I Guideline for Preventing Atherosclerosis in Childhood and Adolescence.^13^ The HOMA-IR index was calculated from the values of glycemia and fasting insulinemia. Due to the different cut-off points found in the literature and their limitations, we adopted the 75^th^ percentile of the HOMA-IR distribution as the cut-off point for the diagnosis of IR because it represents the extreme of the HOMA-IR distribution,^14^ ^,^ ^15^ which is equivalent to 2.27 in the sample.

Food consumption was assessed using a single 24-hour dietary recall, which is a simple, fast, low-cost method that provides mean estimates of energy and nutrient intake. The questionnaire was administered in a face-to-face interview using the “Multiple-Pass Method.”^16^ Dietary variables were collected with a specific software developed for the direct entry of information into netbooks. Consumption was estimated using the Nutritional Composition Table of Food Consumption in Brazil^17^ and the Table of Referred Measures for Food Consumption in Brazil.^18^ Quartiles (25^th^, 50^th^, and 75^th^ percentiles) of energy and macronutrient intake were considered for statistical analysis.

Sexual maturation was self-reported and identified through indicative figures of the stages of sexual maturation proposed by Tanner,^19^ which are divided into three categories: Stage I - pre-puberty; Stages II, III, and IV - puberty; and Stage V - post-puberty.

The monthly frequency of tobacco and alcohol consumption and the physical activity level were investigated to collect behavioral data associated with metabolic risk. Smoking was defined as the use of one or more cigarettes in the 30 days prior to the interview, and binge drinking was established as the consumption of five or more shots of alcoholic beverages on a single occasion in the 30 days prior to the survey.^20^ Adopting the recommendations of the International Physical Activity Questionnaire (IPAQ), we classified the adolescents as “physically active” (those who reported practicing at least 60 minutes of moderate to vigorous physical activities five or more days per week) or “insufficiently active” (those who did not meet the criteria above).^21^

As sampling for the ERICA study involved stratification, clustering, and unequal probabilities,^6^ we performed the statistical analyses using the STATA software, version 14.0, adjusting the complex sampling design with the “survey” module. The explanatory variables were grouped into four hierarchically ordered levels: 1) socioeconomic factors; 2) behavioral factors; 3) individual factors (anthropometric and biochemical characteristics); 4) dietary factors, the latter of which was considered the most proximal level of the model. We determined the conceptual model assuming that factors predisposing to IR imply different hierarchical levels of outcome determination. Initially, we conducted a bivariate analysis to determine associations between IR and the independent variables using simple Poisson regression. Variables with a p<0.20 were incorporated into Poisson multivariate regression models with robust variance adjustment. Results were expressed as prevalence ratios and their respective 95% confidence intervals. Variables with p≤0.05 in the final model were considered statistically associated with IR.

## RESULTS

The final sample was representative of 99,221 adolescents aged 12 to 17 years enrolled in the morning period of public and private schools of Recife. The sample comprised a higher percentage of public-school students (60.1%) and those still in elementary school (53%). The male gender accounted for 50.4% of the sample, and 50.9% were between 12 and 14 years of age (median: 14 years; interquartile range - IQR: 13 to 16 years). Students with lower socioeconomic status (72.1%), whose ethnicity was classified as non-white (73.2%), and whose mother had more than eight years of schooling (70.8%) were predominant.

More than half of the adolescents evaluated were in the pubertal stage of sexual maturation (67.6%), and the others were in the post-pubertal stage (32.4%). Non-smokers (97.8%) and those who did not consume alcoholic beverages (95.8%) were predominant, and 54.9% of the individuals were considered physically active.

IR was confirmed in 25.3% of the population and was more common in females, adolescents aged between 12 and 14 years, those who did not consume alcoholic beverages, and individuals classified as physically inactive. Table 1 displays the distribution of IR according to demographic, socioeconomic (Level 1), and behavioral (Level 2) factors. Table 2 presents the association between the HOMA-IR index and both anthropometric and biochemical variables (Level 3). We found significant associations for all variables analyzed, except LDL-C. Concerning energy and macronutrient intake (Level 4), we identified inverse associations between IR and most food components in the unadjusted analysis (Tables 3 and 4).

**Table 1:** Prevalence of insulin resistance in adolescents according to demographic, socioeconomic, and behavioral factors. Erica-Recife, 2013-2014.

Variables	Insulin Resistance			PR (95%CI)	p-value
	n_observed_	n_estimated_	%		
Level 1 - Demographic/socioeconomic					
Gender					
Male	89	11498	23.0	Ref.	0.156*
Female	182	13668	27.8	1.21 (0.92, 1.57)	
Age range					
15-17 years	100	9730	20.0	Ref.	0.001†
12-14 years	171	15435	30.6	1.53 (1.21, 1.93)	
Ethnicity					
White	67	6353	23.8	Ref.	0.393
Others	201	19151	26.4	1.10 (0.87, 1.40)	
Sexual maturation					
Post-puberty	86	7668	23.8	Ref.	0.597
Puberty	184	17445	26.0	1.09 (0.78, 1.52)	
Maternal schooling					
>8 years	140	17045	24.3	Ref.	0.677
≤8 years	65	7593	26.2	1.08 (0.74, 1.57)	
Socioeconomic status					
High	49	6608	23.9	Ref.	0.956
Low	136	17210	24.1	1.01 (0.73, 1.38)	
Level 2 - Behavioral					
Smoking					
Yes	3	487	22.5	Ref.	0.827
No	268	24782	25.5	1.13 (0.35, 3.59)	
Alcohol consumption					
Yes	9	529	12.6	Ref.	0.121*
No	240	23939	25.2	1.98 (0.82, 4.79)	
Physical activity level					
Active	118	12345	22.7	Ref.	0.178*
Inactive	137	12542	28.0	1.23 (0.90, 1.68)	

PR: prevalence ratio; 95%CI: 95% confidence interval; *Ref:* reference (1.00); *p<0.20; †p<0.05 (simple Poisson regression).

**Table 2:** Prevalence of insulin resistance in adolescents according to anthropometric and biochemical factors. Erica-Recife, 2013-2014.

Variables	Insulin Resistance			PR (95%CI)	p-value
	n_observed_	n_estimated_	%		
Level 3 - Anthropometric/Biochemical					
BMI/A					
Non-overweight	122	10935	15.5	Ref.	<0.001‡
Overweight	73	6536	35.4	2.28 (1.65, 3.15)	
Obesity	76	7695	73.7	4.74 (3.53, 6.36)	
WC					
Normal	202	17776	20.1	Ref.	<0.001‡
High	69	7433	68.5	3.40 (2.61, 4.43)	
WHtR					
Normal	176	16074	19.0	Ref.	<0.001‡
Abdominal obesity	95	9135	62.3	3.27 (2.37, 4.52)	
Total cholesterol					
Desirable	122	11906	21.8	Ref.	0.077*
Borderline	73	6996	28.2	1.29 (0.92, 1.79)	
High	76	6264	31.6	1.45 (1.03, 2.01)	
LDL-C					
Desirable	184	17525	24.1	Ref.	0.542
Borderline	74	6497	29.7	1.23 (0.84, 1.80)	
High	13	1150	24.5	1.01 (0.61, 1.68)	
HDL-C					
Desirable	105	8526	17.1	Ref.	<0.001‡
Low	166	16639	33.7	1.97 (1.54, 2.50)	
TG					
Desirable	171	15127	18.6	Ref.	<0.001‡
Borderline	41	4201	44.7	2.39 (1.79, 3.20)	
High	59	5837	67.3	3.61 (2.84, 4.58)	

PR: prevalence ratio; CI: 95% confidence interval; BMI/A: body mass index-for-age; *Ref:* reference (1.00); WC: waist circumference; WHtR: waist-to-height ratio; LDL-C: low-density lipoprotein cholesterol; HDL-C: high-density lipoprotein cholesterol; TG: triglycerides; *p<0.20; †p<0.05; ‡p<0.001 (simple Poisson regression).

**Table 3:** Prevalence of insulin resistance in adolescents according to energy and carbohydrate intake. Erica-Recife, 2013-2014.

Variables	Insulin Resistance			PR (95%CI)	p-value
	n_observed_	n_estimated_	%		
Level 4 - Food consumption					
Energy intake (kcal)					
<1761.44	89	8953	38.3	2.17 (1.56, 3.01)	<0.001†
1761.44-2422.74	71	5755	24.7	1.40 (0.93, 2.11)	
2422.74-3237.17	63	5713	22.3	1.26 (0.91, 1.73)	
>3237.17	48	4744	17.6	Ref.	
Carbohydrates (g)					
<230.8	90	8417	34.9	1.88 (1.33, 2.67)	<0.001†
230.8-320.8	72	6303	27.1	1.46 (0.93, 2.28)	
320.8-434.5	51	4798	18.5	Ref.	
>434.5	58	5647	21.7	1.17 (0.76, 1.79)	
Refined sugar (g)					
<86.1	76	7495	29.3	1.46 (0.97, 2.21)	0.097*
86.1-137.3	83	7272	28.5	1.42 (0.99, 2.03)	
137.3-195.4	59	5383	23.4	1.17 (0.67, 2.03)	
>195.4	53	5015	20.0	Ref.	
Added sugar (g)					
<60.8	80	8308	32.9	1.61 (1.07, 2.40)	0.126*
60.8-104.4	68	6188	24.7	1.21 (0.85, 1.72)	
104.4-163.0	71	5646	23.2	1.13 (0.70, 1.82)	
>163.0	52	5024	20.4	Ref.	

PR: prevalence ratio; CI: 95% confidence interval; *Ref.:* reference (1.00); *p<0.20; †p<0.05; ‡p<0.001 (simple Poisson regression).

**Table 4:** Prevalence of insulin resistance in adolescents according to dietary fat intake. Erica-Recife, 2013-2014.

Variables	Insulin Resistance			PR (95%CI)	p-value
	n_observed_	n_estimated_	%		
Level 4 - Food consumption					
Total lipids (g)					
<54.5	83	8369	35.6	1.76 (1.20, 2.57)	0.028†
54.5-80.3	79	6320	26.4	1.30 (0.89, 1.92)	
80.3-115.9	55	5371	20.3	1.01 (0.65, 1.55)	
>115.9	54	5105	20.2	Ref.	
Saturated fat (g)					
<18.8	82	8015	34.5	1.83 (1.21, 2.78)	0.032†
18.8-27.9	71	6384	25.9	1.37 (0.86, 2.21)	
27.9-42.9	67	5892	23.2	1.23 (0.80, 1.89)	
>42.9	51	4874	18.7	Ref.	
Polyunsaturated fat (g)					
<8.7	82	7726	33.8	1.53 (1.01, 2.30)	0.199*
8.7-13.5	70	5523	23.3	1.05 (0.69, 1.60)	
13.5-22.0	63	6229	23.1	1.04 (0.72, 1.50)	
>22.0	56	5687	22.1	Ref.	
Omega-6 (g)					
<7.6	81	7601	33.3	1.51 (0.98, 2.32)	0.235
7.6-11.8	70	5651	23.7	1.07 (0.75, 1.51)	
11.8-19.2	63	5916	22.1	Ref.	
>19.2	57	5997	23.3	1.05 (0.71, 1.55)	
Omega-3 (g)					
<0,9	78	7331	32.8	1.52 (1.01, 2.29)	0.221
0.9-1.4	64	5284	21.5	Ref.	
1.4-2.3	73	6771	24.7	1.14 (0.73, 1.79)	
>2.3	56	5779	23.1	1.07 (0.70, 1.62)	
Monounsaturated fat (g)					
<17.5	87	8346	35.7	1.86 (1.27, 2.71)	0.027†
17.5-26.4	72	5989	24.9	1.29 (0.91, 1.85)	
26.4-38.9	62	5878	22.6	1.17 (0.77, 1.77)	
>38.9	50	4951	19.2	Ref.	
Dietary cholesterol (g)					
<140.1	69	6920	30.0	1.35 (0.81, 2.26)	0.484
140.1-228.3	76	6799	27.3	1.23 (0.88, 1.71)	
228.3-396.4	58	5723	22.5	1.01 (0.71, 1.43)	
>396.4	68	5724	22.1	Ref.	
Trans fat (g)					
<1.0	74	7290	28.6	1.33 (0.96, 1.84)	0.353
1.0-1.7	69	6137	25.3	1.18 (0.92, 1.52)	
1.7-3.1	68	6393	26.0	1.21 (0.82, 1.79)	
>3.1	60	5345	21.4	Ref.	

PR: prevalence ratio; CI: 95% confidence interval; *Ref.:* reference (1.00); *p<0.20; †p<0.05; ‡p<0.001 (simple Poisson regression).

After the statistical adjustments performed according to the pre-established hierarchical model, the variable that remained significantly associated with IR on Level 1 was age range; none of the variables on Level 2 were significantly associated with the outcome; on Level 3, the anthropometric index BMI/A and the biochemical markers TG and HDL-C remained statistically significant; and on Level 4, energy intake, total lipids, polyunsaturated fat, and monounsaturated fat had a statistically significant association with the outcome (Table 5).

**Table 5:** Adjusted prevalence ratios of insulin resistance in adolescents according to explanatory variables. Erica-Recife, 2013-2014.

Levels/variables	Insulin resistance				p-value
	Crude analysis		Adjusted analysis		
	PR	95%CI	PR	95%CI	
Level 1 - Demographic/socioeconomic					
Age range					
15-17 years	Ref.		Ref.		
12-14 years	1.53	(1.21, 1.93)	1.54	(1.23, 1.95)	0.001*
Level 3 - Anthropometric/biochemical					
BMI/A					
Non-overweight	Ref.		Ref.		
Overweight	2.28	(1.65, 3.15)	1.92	(1.34, 2.75)	0.001*
Obesity	4.74	(3.53, 6.36)	3.14	(2.22, 4.46)	<0.001†
TG					
Desirable	Ref.		Ref.		
Borderline	2.39	(1.79, 3.20)	1.67	(1.23, 2.26)	0.002*
High	3.61	(2.84, 4.58)	2.13	(1.71, 2.67)	<0.001†
HDL-C					
Desirable	Ref.		Ref.		
Low	1.97	(1.54, 2.50)	1.51	(1.0, 2.28)	0.050*
Level 3 - Food consumption					
Energy intake (kcal)					
<1761.44	2.17	(1.56, 3.01)	4.23	(1.32, 13.5)	0.016*
1761.44-2422.74	1.40	(0.93, 2.11)	2.79	(0.93, 8.29)	0.064
2422.74-3237.17	1.26	(0.91, 1.73)	2.35	(1.11, 4.99)	0.027*
>3237.17	Ref.		Ref.		
Total lipids (g)					
<54.5	1.76	(1.20, 2.57)	0.53	(0.17, 1.61)	0.256
54.5-80.3	1.30	(0.89, 1.92)	0.71	(0.28, 1.78)	0.463
80.3-115.9	1.01	(0.65, 1.55)	0.40	(0.17, 0.91)	0.032*
>115.9	Ref.		Ref.		
Polyunsaturated fat (g)					
<8.7	1.53	(1.01, 2.30)	0.66	(0.38, 1.15)	0.144
8.7-13.5	1.05	(0.69, 1.60)	0.57	(0.37, 0.87)	0.012*
13.5-22.0	1.04	(0.72, 1.50)	0.92	(0.60, 1.42)	0.718
>22.0	Ref.		Ref.		
Monounsaturated fat (g)					
<17.5	1.86	(1.27, 2.71)	1.40	(0.52, 3.78)	0.488
17.5-26.4	1.29	(0.91, 1.85)	1.05	(0.40, 2.73)	0.914
26.4-38.9	1.17	(0.77, 1.77)	1.78	(1.02, 3.08)	0.040*
>38.9	Ref.		Ref.		

PR: prevalence ratio; CI: 95% confidence interval; *Ref.:* reference (1.00); BMI/A: body mass index-for-age; TG: triglycerides; HDL-C: high-density lipoprotein cholesterol; *p≤0.05; †p<0.001 (Poisson regression with robust variance adjustment). Level 1 adjusted for stage of sexual maturation. Level 3 adjusted for variables of level 1 and stage of sexual maturation. Level 4 adjusted for variables of levels 1 and 3 and stage of sexual maturation.

## DISCUSSION

Studies addressing the association between IR (measured using the HOMA-IR index) and variables related to metabolic outcomes and food consumption among Brazilian adolescents are scarce in the literature. Research usually focuses on the occurrence of metabolic syndrome and its associated factors, which is an event frequently observed in these individuals, with rates ranging from 3.4 to 45.5%, depending on the diagnostic criteria.^1^ ^,^ ^22^

Researchers recommend the use of the HOMA-IR index to assess IR in epidemiological investigations.^5^ ^,^ ^23^ However, there is no consensus in the literature regarding the cut-off point to use for adolescents, which leads to variations in the prevalence of IR reported.^1^ ^,^ ^5^ ^,^ ^24^ In the present study, we adopted the 75^th^ percentile of the HOMA-IR index, which corresponded to 2.27. This cut-off point is lower than the 3.16 recommended by I Guideline for Preventing Atherosclerosis in Childhood and Adolescence,^13^ which is frequently used in Brazilian studies reporting similar or lower prevalence rates of IR in comparison to the present investigation (10 to 29%).^1^ ^,^ ^22^ ^,^ ^25^

In a study conducted by Li et al.^24^ involving American adolescents aged between 12 and 19 years who participated in the 2005‒2006 National Health and Nutrition Examination Survey (NHANES), the use of the 75^th^ percentile of the HOMA-IR index led to a lower prevalence rate of IR when compared to the one found in the present sample (8.7 vs. 25.3%). We emphasize, however, that the NHANES sample was representative of the entire United States, and the population in the present study was representative only of the capital of the state of Pernambuco. Moreover, the authors cited did not report the cut-off point corresponding to 75^th^ percentile of the index.

Analyzing a representative sample of 2,716 Korean adolescents between the ages of 10 and 20 years, using the 95^th^ percentile of the HOMA-IR, Yi et al.^5^ found a lower prevalence rate of IR (9.8%), which was higher among males (10.9%) than females (8.6%). In contrast, this disorder affected more female adolescents in the present study, which is in agreement with the findings of other studies, despite the different cut-off points used for the HOMA-IR index.^1^ ^,^ ^22^ ^,^ ^23^ The distinction in distribution between genders is due to differences in the changes in body composition between boys and girls and the action of hormones characteristic of the sexual maturation phase.^22^ However, we found no significant association between the stages of sexual maturation and the HOMA-IR in the present study. On the other hand, younger individuals (12 to 14 years) had a greater frequency of IR, and this association remained significant after the statistical adjustments. The occurrence of this event in younger individuals, probably in the early stages of puberty, may be explained by the fact that IR increases as a physiological response to puberty and the advance in age, returning to baseline levels after the growth spurt.^1^

In the unadjusted analysis, adolescents who did not drink alcohol were more affected by IR. However, this finding may have been due to the large number of non-drinkers in the sample. Although the consumption of moderate amounts of alcohol can lead to an improvement in insulin sensitivity in adults,^26^ this behavior should not be adopted as a measure for improving metabolic syndrome in adolescents.

A higher percentage of physically inactive individuals had a diagnosis of IR. This fact is worrisome, since a sedentary lifestyle contributes directly to weight gain, which is a primary risk factor for IR.^5^ In contrast, the regular practice of physical activity of moderate intensity can enhance the insulin response for up to 48 hours after physical training,^27^ help maintain an ideal body weight, and improve both physical and psychological quality in adolescence and adulthood.

Overweight and obese adolescents had a threefold higher prevalence rate of IR compared to non-overweight adolescents. According to Lavrador et al.,^28^ IR is strongly associated with the severity of obesity, and a dose-response gradient can be inferred from this association.

The type of body fat distribution can also determine the development of IR, with greater risk observed mainly among individuals with fat accumulated in the visceral or abdominal region, which concentrates a larger amount of cells that activate the inflammatory state, thereby contributing to IR.^26^ In a cross-sectional study using the 75^th^ percentile as the cut-off point for 486 children and adolescents aged 5 to 15 years, Burrows et al.^29^ found a direct association between the HOMA-IR index and WC; the authors also identified inadequate levels of TG and HDL-C, which is in agreement with the present findings. When assessing Brazilian adolescents between 10 and 19 years of age with HOMA-IR subdivided in percentiles, Rocco et al.^30^ found that a higher percentile of the index denoted a greater risk for the other metabolic variables analyzed. Likewise, Gobato et al.^1^ analyzed the distribution of the HOMA-IR in tertiles and verified that the mean of all body composition indicators assessed in the study, including BMI and WC, increased with the rise in HOMA-IR scores.

The associations found between the HOMA-IR index and both TG and HDL-C in the adjusted analysis may have occurred because overweight and obesity increase the number of adipocytes, consequently releasing free fatty acids into the bloodstream.^26^ Moreover, IR raises the serum levels of LDL-C, resulting in reduced HDL-C concentrations, the activation of which depends on the degradation of LDL-C.^1^

Our study demonstrated an inverse association between energy intake and IR. Regarding the consumption of total and polyunsaturated fats, we identified a protective effect. In contrast, the consumption of monounsaturated fats did not show benefits in terms of lower prevalence of the outcome, similar to the study by Kahleova et al., in 2019.^4^

Epidemiological data regarding the influence of diet on IR in adolescents are scarcely available in the literature, and this issue requires further clarification. In a cross-sectional study comprising 100 female adolescents aged between 12 and 14 years, enrolled in public schools in the city of Viçosa (Southeastern Brazil), Faria et al.^25^ found a higher intake of saturated fat among adolescents without metabolic syndrome, which was the outcome analyzed. According to the authors, this finding may have been due to information bias, as overweight adolescents often underestimate their current consumption of fats, or reverse causality (e.g., overweight and obese adolescents may have been on a diet to lose weight). Indeed, these factors must also be taken into account when interpreting the dietary data reported in the present investigation. Nonetheless, the harmful effects of the excessive consumption of foods rich in refined sugar as well as saturated and trans fats are well established in the literature.^3^ ^,^ ^4^ ^,^ ^25^ Inadequate eating habits combined with physical inactivity favor overweight and the establishment of a pro-inflammatory condition that contributes to the development of IR.^3^ ^,^ ^4^

The present study involved a representative sample of adolescents and described data from a place with no previous research on the subject. However, some limitations should be considered. The cross-sectional design does not allow us to determine cause-and-effect relationships. The administration of just one 24-hour dietary recall only reveals the current consumption, which also renders the determination of causality impossible. Moreover, the use of self-reported data may increase the risk of information bias. Another factor to consider was the different cut-off points used for the HOMA-IR in the articles consulted, which hinders the comparison of results.

In short, the present findings reveal that IR was prevalent in the population analyzed. This is an issue of concern, as the sample consisted of individuals in the early stages of life. The associations between the HOMA-IR index and variables related to metabolic outcomes enabled the recognition of the factors most connected to IR, which can assist in the implementation of public health strategies for preventing this disorder in adolescents by strengthening actions directed at addressing predisposing conditions, especially overweight. Further studies on food consumption in this age group are necessary and should take into account eating behaviors and habits in a more global way to allow better recognition of food characteristics that pose a risk for the development of IR and metabolic disorders in adolescence.
